# Supporting Older Family Caregivers of Young Adults with IDD: A Pilot Program with Socially Assistive Robotics

**DOI:** 10.1093/geroni/igab046.2971

**Published:** 2021-12-17

**Authors:** Ling Xu, Julienne Greer, Noelle Fields, Priscila Tamplain, John Bricout, Bonita Sharma, Kristen Doelling

**Affiliations:** 1 University of Texas at Arlington, Arlington, Texas, United States; 2 The University of Texas at Arlington, Arlington, Texas, United States; 3 University of Minnesota, twin cities, Minnesota, United States; 4 UT-San Antonio, San Antonio, Texas, United States; 5 UTA, Arlington, Texas, United States

## Abstract

Introduction: The need for caregiver respite is well-documented for the care of persons with IDD. Social Assistive Robotics (SAR) offer promise in addressing the need for caregiver respite through ‘complementary caregiving’ activities that promote engagement and learning opportunities for a care recipient (CR) with IDD. This study explored the acceptability and usefulness of a SAR caregiver respite program responsive to feedback from both the CRs and their older family caregivers (age 55+). Methods: Caregiver-CR dyads (N =11) were recruited. A mixed methods research design was deployed in three phases: Phase I with four focus groups to inform the program design; Phase II for program demonstration and evaluation with pre- and post-surveys; and Phase III with post-program interviews for feedback and suggestions. Results: Quantitative data analysis in Phase II showed both caregivers and their CRs scored favorably the social presence of the robot (Pepper) and social engagement with Pepper. Caregivers also reported positive perceptions of Pepper in terms of anthropomorphism, animation, likeability, intelligence, and safety. Content analysis from Phase III interviews suggested that the SAR may offer physical/emotional respite to caregivers by providing companionship/friendship as well as promoting independence, safety/monitoring, and interactive engagement with children. Discussion: SAR has potential in providing respite for older family caregiver demonstrated by the levels of CR engagement and learning with Pepper. Future studies need a longer program design and larger sample size to test the feasibility and efficacy of the intervention.

